# Investigations on the Optical Properties of InGaN/GaN Multiple Quantum Wells with Varying GaN Cap Layer Thickness

**DOI:** 10.1186/s11671-020-03420-y

**Published:** 2020-10-01

**Authors:** Xiaowei Wang, Feng Liang, Degang Zhao, Zongshun Liu, Jianjun Zhu, Jing Yang

**Affiliations:** 1 State Key Laboratory of Integrated Optoelectronics, Institute of Semiconductors, Chinese Academy of Sciences, Beijing, 100083 China; 2 College of Materials Science and Opto-Electronic Technology, University of Chinese Academy of Sciences, Beijing, 100049 China

**Keywords:** GaN cap layer, Piezoelectric field, Localization states

## Abstract

Three InGaN/GaN MQWs samples with varying GaN cap layer thickness were grown by metalorganic chemical vapor deposition (MOCVD) to investigate the optical properties. We found that a thicker cap layer is more effective in preventing the evaporation of the In composition in the InGaN quantum well layer. Furthermore, the quantum-confined Stark effect (QCSE) is enhanced with increasing the thickness of GaN cap layer. In addition, compared with the electroluminescence measurement results, we focus on the difference of localization states and defects in three samples induced by various cap thickness to explain the anomalies in room temperature photoluminescence measurements. We found that too thin GaN cap layer will exacerbates the inhomogeneity of localization states in InGaN QW layer, and too thick GaN cap layer will generate more defects in GaN cap layer.

## Introduction

InGaN/GaN multiple quantum wells (MQWs) structure is widely used in visible light-emitting diodes (LEDs) and laser diodes (LDs) as an active region, and has achieved great success [1–4]. Improving the quality of InGaN/GaN MQWs is technologically important for realizing high-performance GaN-based LEDs and LDs. In previous research, it was found that high-temperature grown GaN (HT-GaN) quantum barriers (QBs) are beneficial to improve the crystal quality and luminous intensity of InGaN/GaN quantum wells [5, 6]. Due to the weak In-N bond strength, the growth temperature of InGaN quantum wells (QWs) layer is generally lower than the GaN QBs to obtain high In content. This may cause the indium atoms to evaporate from InGaN QW during the growth of GaN QB at higher temperature. Inserting a low-temperature-grown GaN cap (LT-GaN cap) layer between InGaN QW and GaN QB has been proven to effectively reduce In composition desorption, which can improve the uniformity of the thickness of InGaN QW layer and the distribution of In composition [7–9]. However, when the growth temperature of GaN cap layer decreases, the migration ability of the adatoms becomes weaker, resulting in the deterioration of GaN materials. Moreover, it is reported that the number of defects increases with increasing low-temperature-grown GaN cap layer thickness, which is not in favor of improving the uniformity of indium atoms distribution and the luminous intensity of InGaN quantum wells [10]. Many previous reports did not focus on the effect of localization states on luminescence properties in active region with various GaN cap thicknesses. In this work, we use X-ray diffraction (XRD), electroluminescence (EL), and photoluminescence (PL) to report the influence of low-temperature-grown GaN cap layer thickness on the structure and the luminescence properties of the InGaN/GaN MQWs, and describe in detail the role of localization states in luminescence characteristics by temperature-dependent PL experiments.

## Methods

Three InGaN/GaN MQWs samples were grown on the c plane sapphire substrate by metalorganic chemical vapor deposition (MOCVD). All samples contain the same structures, consisting of an undoped GaN buffer layer (1.2 μm), a Si-doped n-GaN layer (1 μm), a two-period InGaN/GaN MQW active region and a Mg-doped p-GaN layer (40 nm). In the process of growing InGaN/GaN active region, the growth conditions of InGaN QWs were identical to each other; a thin LT-GaN cap layer was grown at the same temperature as InGaN QW (710 °C) to prevent indium evaporating, and the growth time of LT-GaN cap layer was 150 s, 300 s, and 500 s for sample A, B and C, respectively; and then the growth temperature of GaN QB was raised up to 810 °C to improve the material quality of GaN QB, and the growth time of HT-GaN QB was identical for these samples. X-ray diffraction (XRD) was used to determine the structural parameters of these three samples. Electroluminescence (EL) and room temperature photoluminescence (PL) were carried out to characterize the optical properties of the active regions.

The EL spectra were measured using Ocean Optics HR2000 high-resolution spectrometer at direct current (DC), where the output power is detected by using a Si photodiode, and the EL spectral peak energy and FWHM are detected by a monochromator and a photomultiplier tube. For PL measurement, a *λ* = 325 nm continuous wave He-Cd laser was used as an excitation source, with an incident optical power of 3 mW and a spot size of 0.5 mm^2^. Temperature-dependent PL measurements from 30 K to 300 K were recorded to analyze the effect of localization states on luminescence characteristics by using a 405 nm GaN semiconductor laser with 5 mW emission power as an excitation source. The sample was cooled by free-flow liquid helium and a closed-cycle refrigerator of CTI Cryogenics was used for the temperature-dependent measurements

## Results and Discussions

**Fig. 1:**
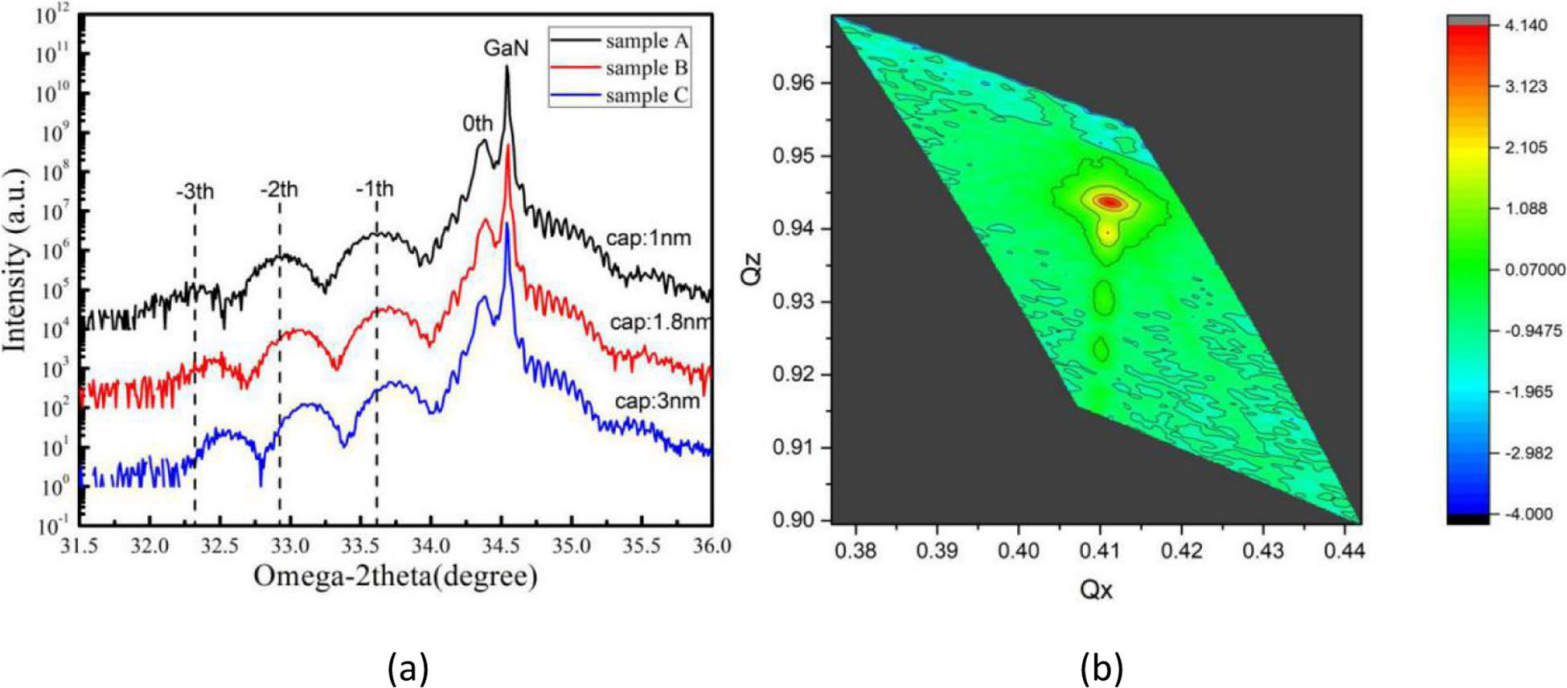
**a** XRD ω-2θ scan curves on GaN (0002) plan for samples A, B, and C. **b** Reciprocal space mapping of sample C

Figure 1a shows the diffraction patterns of the (0002) plane taken by ω-2θ scan for these samples. The satellite peaks of these samples can be clearly seen, indicating that fine periodic structure and sharp interface exist in these MQWs. We use SmartLab Studio II and Global Fit program (offering advanced parallel tempering base fitting method to analyze the data generated by the SmartLab diffractometer) to obtain the average In composition, and QB and QW thickness, as shown in Table 1. It can be clearly seen that the In composition in the QW increase with increasing the thickness of LT-GaN cap layer, indicating that a thicker cap layer is more effective in preventing the evaporation of the In composition. Figure 1b shows the reciprocal space mapping (RSM) of sample C. The main GaN peak and satellite peaks are sit on a line, suggests that the QW layers with the highest In content in all samples are completely in strain. Therefore, the influence of piezoelectric field on luminous properties should be taken into consideration.

**Table 1:** Structural parameters of InGaN/GaN MQWs of samples A, B, and C determined by XRD measurements

Sample	Thickness (nm)			In content (%)	
	QW	QB	Cap	QW	QB
A	4.0	11.1	1.0	10.8	0.00
B	4.0	11.0	1.8	11.9	0.00
C	4.0	11.0	3.0	13.6	0.00

**Fig. 2:**
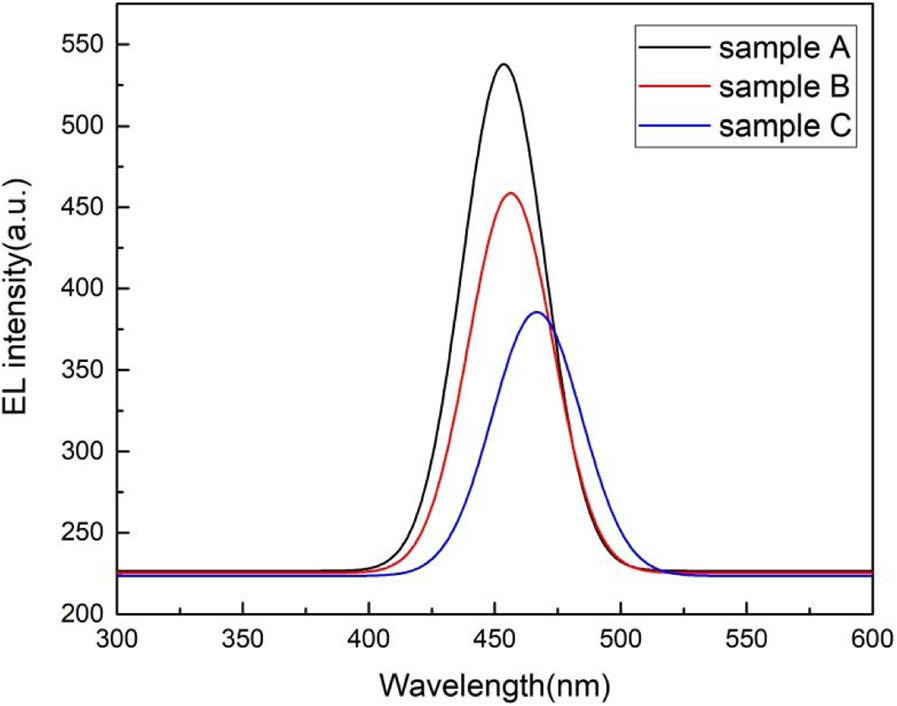
EL spectra of samples A, B, and C under 5 mA current

**Fig. 3:**
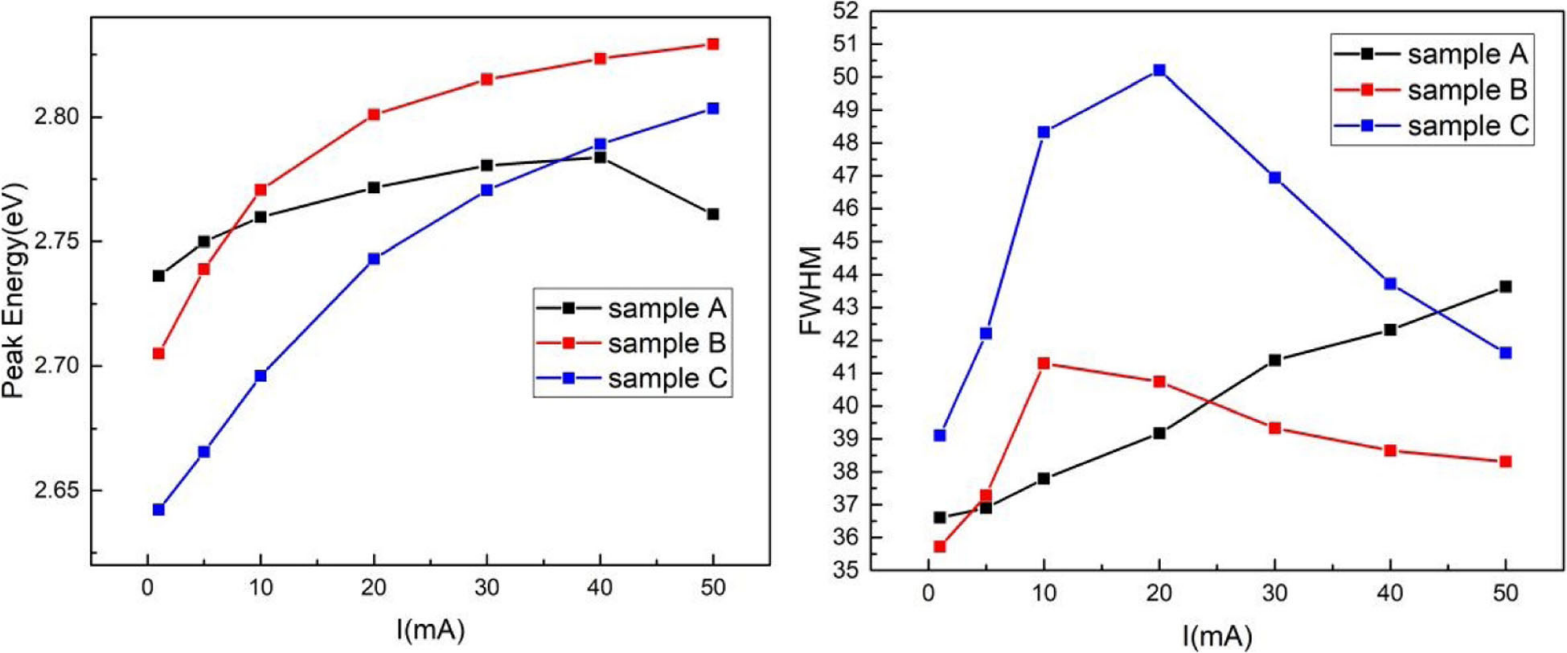
(**a**) EL peak energy and (**b**) EL full width at half maximum (FWHM) as a function of injection current for samples A, B, and C

Figure 2 shows the EL spectra of samples A, B, and C under 5 mA current. The peak wavelength is 453.6 nm, 456.3 nm, and 466.7 nm for samples A, B, and C, respectively. From the results of XRD measurements, as the thickness of the LT-GaN cap layer increases, the increased In composition in QWs will induce the EL peak energy red shift. We also found the EL intensity decrease with increasing LT-GaN cap layer thickness. It is known that a thicker GaN barrier layer (the total thickness of LT-GaN cap layer and HT-GaN QB) increases the distance of holes from p-GaN to the active region, resulting in a reduction in hole injection efficiency, which leads to a decrease in EL intensity [11, 12]. In addition, the increase of In composition will enhance the piezoelectric field in InGaN QW layer, so that the energy band tilt is aggravated, resulting in a red-shift of peak emission and a reduced luminous efficiency, which is known as quantum-confined Stark effect (QCSE) [13–15]. To verify the influence of QCSE on EL, peak energy, and FWHM of EL changing with different injection are shown in Fig. 3. As the injection current increases, the emission peak energy blue shift can be ascribed to the band-filling effect and QCSE compensated by the electron screening effect [16–18]. Since sample C with the most In composition has the deepest potential well and strongest piezoelectric field, the band-filling effect and charge screening effect are the most significant, so the amount of EL peak energy blue shift is the largest for sample C (166 meV). The band-filling effect and charge screening effect in sample A is the weakest, so that the band-gap shrinking owing to thermal effect is gradually dominant under high injection current (50 mA), resulting in a red-shift of peak energy. For sample A, the FWHM increases with increasing injection current as shown in Fig. 3b, indicating that EL spectrum broadening induced by the band-filling effect is always dominant. It has been reported that charge screening of polarized electric field in the InGaN QWs will cause the narrowing of EL spectral bandwidth with increasing injection current [19]. Therefore, for samples B and C, the band-filling effect is significant at lower injection currents, and the EL spectrum is broadened. As the injection current increases further, carriers screening of polarized electric field process gradually takes over, so the FWHM declines. In addition, we also found that the current corresponding to the turning point of FWHM from rising to falling for samples B and C is about 10 mA and 20 mA respectively, which also implies that the piezoelectric field in sample C is the strongest.

**Fig. 4:**
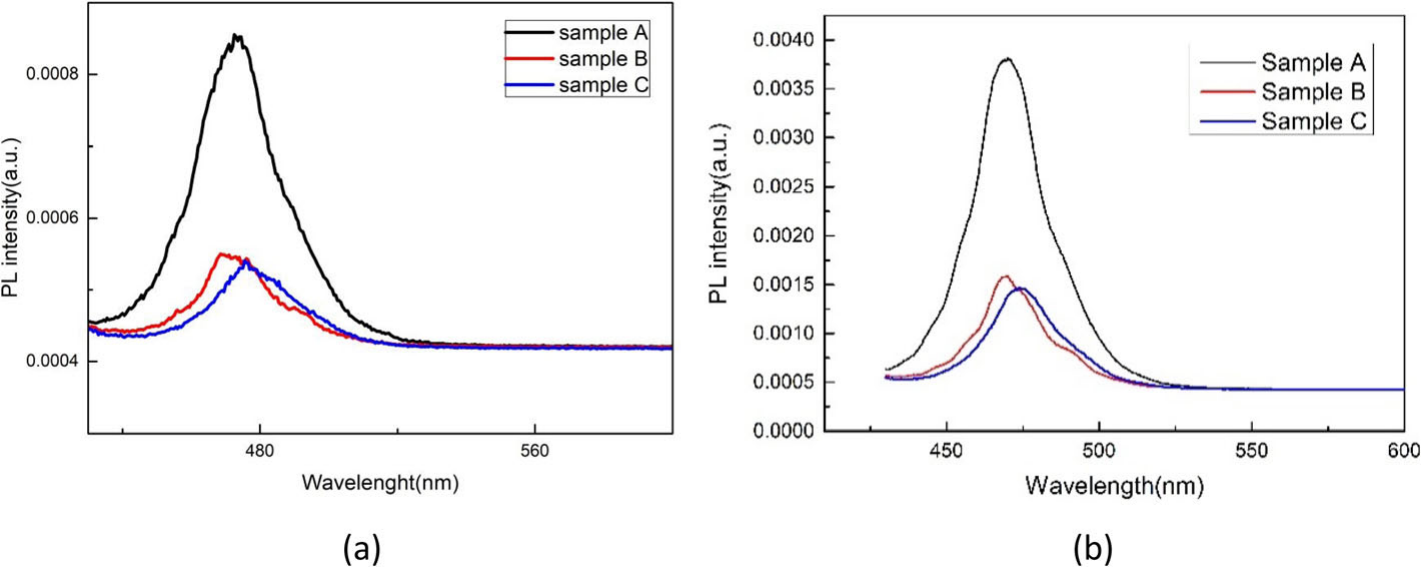
Room-temperature PL spectra for samples A, B, and C by using a 405 nm GaN semiconductor laser (**a**) and 325 nm He-Cd laser (**b**). The peak wavelength is 473.1 nm, 472.9 nm, and 478.2 nm obtained from (**a**) and 470.5 nm, 470.1 nm, and 475.2 nm obtained from (**b**) for samples A, B, and C, respectively

**Fig. 5:**
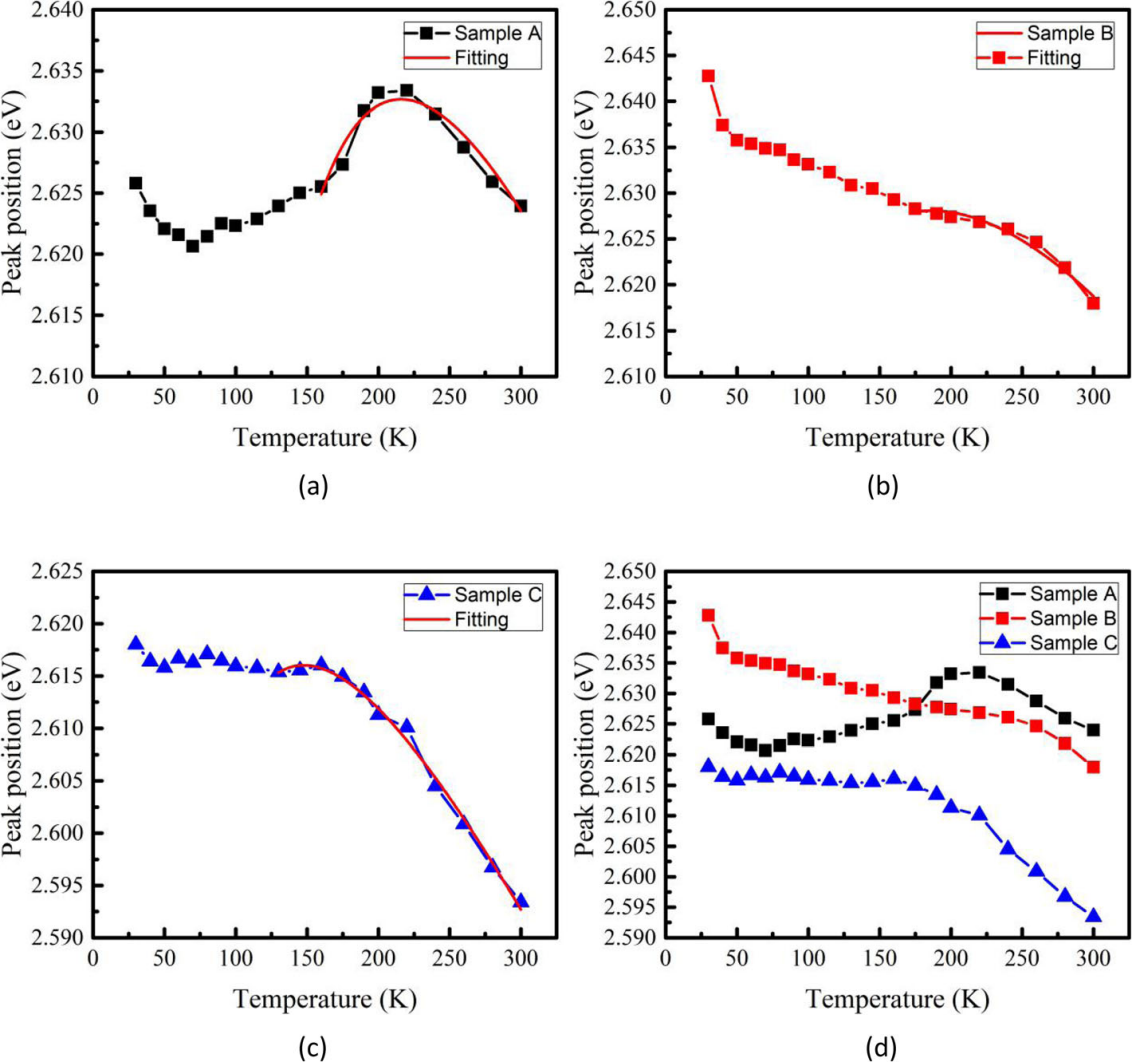
The red lines in (**a**), (**b**), and (**c**) are fitting curves based on Eq. (1) and the fitting parameter *σ* is 36.96 meV, 18.89 meV, and 23.77 meV for samples A, B, and C, respectively. (**d**) Shows the experimental data of PL peak energy as a function of temperature

Figure 4a shows the room temperature PL spectra for samples A, B, and C at 5 mW 405 nm GaN semiconductor laser excitation power. The peak wavelength is 473.1 nm, 472.9 nm, and 478.2 nm for samples A, B, and C, respectively. Since the PL intensity of samples B and C in Fig. 4a are relatively weak, we also utilize 325 nm He-Cd laser as an excitation source to execute the photoluminescence experiment. The PL experimental results in Fig. 4a and b are consistent with each other, which eliminates the uncertainty of the experimental results obtained from Fig. 4a. Compared with the EL measurement results from Fig. 2, we found some anomalies in Fig. 4a: (1) Sample A contains less In composition than sample B, but the peak wavelength of sample A is slightly longer than sample B; (2) the PL intensity of sample B and C is similar to each other, and the luminous intensity of sample A is much higher than sample B and C. To find out the relevant reasons to explain these anomalies, temperature-dependent PL (TDPL) was performed for three samples, and the PL peak energy as a function of temperature is shown in Fig. 5. For sample B, the emission peak energy monotonically decreases with increasing temperature. Generally speaking, increasing temperature-induced band gap shrinkage will result in a red shift of emission energy. However, there is a notable blue shift in sample A when temperature increases from 70 K to 190 K. The thermal redistribution of localized excitons has been introduced to explain the abnormal emission blue shift in TDPL: with temperature rising, localized excitons were thermally activated and thermally transfer from potential minima of localization states to a higher energy state [5, 20, 21]. For sample C, PL peak energy is almost unchanged in the range of 50 K to 175 K, indicating the band gap shrinkage effect and excitons localization effect offset each other.

Considering the exciton localization effect, the band-tail model can be revised to the following expression to describe the temperature-dependent emission peak energy [22, 23]:

1 $$ {E}_0(T)={E}_0(0)\hbox{-} \frac{\alpha {T}^2}{T+\beta}\hbox{-} \frac{\sigma^2}{{\mathrm{k}}_BT} $$

where *E*_0_(0) is the band gap at T = 0, *α* and *β* are Varshini’s parameters, *k*_B_ is the Boltzmann constant. The third term *σ* is the standard deviation of the localized distribution and describes the degree of localization effect, i.e., a larger value of *σ* means a stronger inhomogeneity of localization states. The fluctuation of In composition in InGaN may be the main reason for the formation of band tail. The states in the band tails are formed at the minimum local potential energy, similar to quantum dots. The recombination of electron-hole pairs in these self-formed quantum dots can be regarded as localized exciton recombination [24, 25]. The model is not applicable at low temperatures because of strong degeneracy and possible deviation from quasi-equilibrium [20]. The fitting parameter *σ* is 36.96 meV, 18.89 meV, and 23.77 meV for samples A, B, and C respectively, which implies that the localization states of sample A with the thinnest cap layer is the most inhomogeneous. In general, localization states originate from In-rich clusters with different sizes and the fluctuation of QW thickness in InGaN alloys. Therefore, we reasonably believe that a thinner LT-GaN cap layer cannot effectively prevent indium evaporating when increasing temperature to a grown GaN QB layer. The process of indium composition random evaporation results in fluctuation of In content and InGaN QW thickness. For sample B, 1.8 nm-thickness GaN cap layer is thick enough to protect In from desorption to form a more uniform InGaN QW layer. Inhomogeneous localization states usually retain a deeper potential state and result in a red shift of emission peak energy. That is why the peak wavelength of sample A is longer than sample B observed from Fig. 4. In addition, an increase of In composition in InGaN QW layer will also promote the formation of large size In clusters, which explains why the exciton localization effect in sample C with the highest indium content is slightly stronger than sample B.

**Fig. 6:**
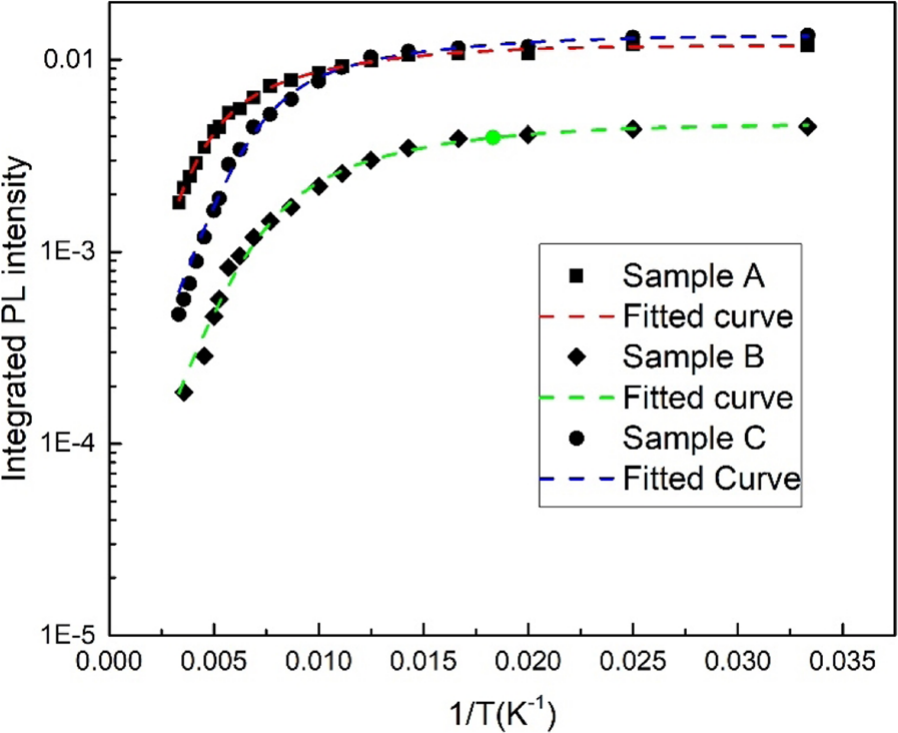
Integrated PL intensity as a function of reverse temperature for samples A, B, and C

Figure 6 shows integrated PL intensity as a function of temperature for sample A and C, which can be well fitted by the following expression [5, 26]:

2 $$ I(T)=\frac{1}{1+{\sum}_{\mathrm{i}}{C}_i\exp \left(-\frac{E_i}{k_BT}\right)} $$

where *C*_i_ is a constant corresponding to the density of non-radiative recombination centers, and *E*_i_ represents the activation energy of non-radiative recombination centers. The fitting parameter *C*_i_ and *E*_i_ were shown in Table 2. It is noticeable that photomultiplier voltage in grating spectrometer for sample B was set lower than samples A and C during temperature-dependent PL measurements; therefore, the integrated PL intensity of sample B is the lowest, but it does not affect fitting results.

**Table 2:** Fitting parameters based on Eq. (2) for samples A, B, and C

Sample	*C*_1_	*E*a_1_ (meV)	*C*_2_	*E*a_2_ (meV)
A	2.95	18	85.25	79.67
B	5.03	16	213.58	69.52
C	2.94	15	220.38	63.32

**Fig. 7:**
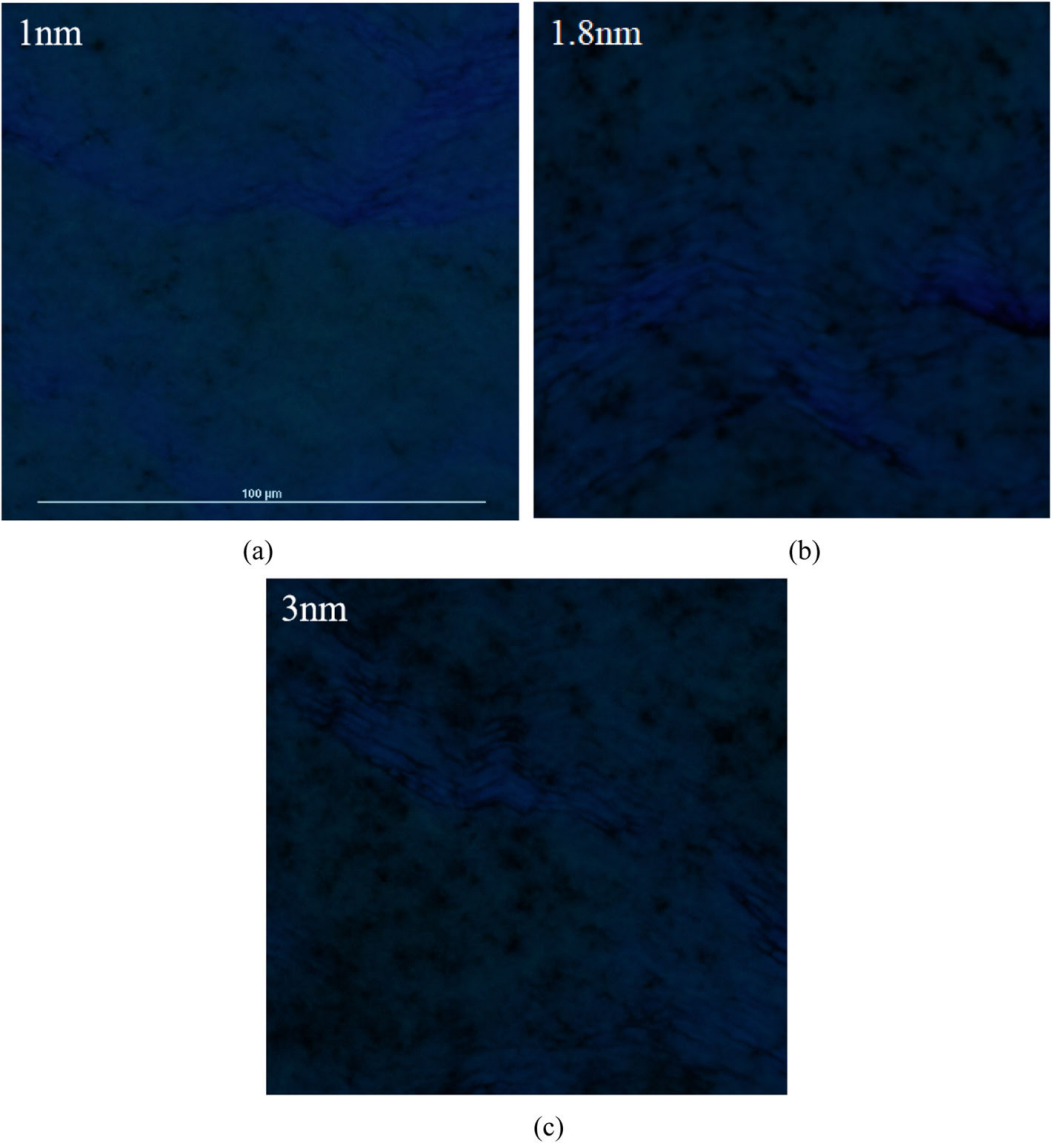
Micro-PL images of samples A, B, and C with various LT-GaN cap layer thickness (**a**) 1 nm, (**b**) 1.8 nm, and (**c**) 3.0 nm

*C*_2_ for samples B and C are larger than sample A, indicating that more defects are formed in a thicker LT-GaN cap layer. It has been observed that the defects in the LT-cap layer increasing with increasing the thickness of the LT-cap layer [6, 10]. The influence of defects in the LT-GaN cap layer next to the quantum well on PL intensity is significant as the electron and hole wave functions penetrate into the barriers. The active energy of 79.67 meV (*E*_a2_ for sample A) is related to the emission of holes from QWs followed by trapping by barrier defects and nonradiative recombination, which consist with the Olaizola’s reports [6]. We also compared the micro-PL images of samples A, B, and C as shown in Fig. 7. The dark spots in micro-PL images can be attributed to the nonradiative recombination in the InGaN/GaN active region. Compared to sample A, more large size dark spots can be obviously observed in samples B and C, indicating that more defects exist in a thicker LT-GaN cap layer. In general, thermal annealing is implemented following the LT cap layer to reduce the indium cluster in the InGaN layer and improve the crystal quality. If the cap layer is too thick, the effect of thermal annealing will be weakened, and the formation of more metallic indium precipitates will hinder the adatoms of GaN cap layer diffusion and introduce more defects in the LT-cap layer. The appearance of more defects and stronger QCSE in InGaN/GaN MQWs with a thicker LT-GaN cap layer is disadvantageous to improve luminescence intensity, so why is the PL intensity of C comparable to B? In fact, localization states also play an important role in improving luminous efficiency. Carriers can be captured into localized states and radiatively recombine. The stronger excitons localized effect, the more carriers will not be captured into defects, which seem to explain the PL intensity of samples B and C is similar to each other, and the luminous intensity of sample A is much higher than samples B and C.

## Conclusions

In summary, the influence of the GaN cap layer thickness on the optical characteristics was investigated. The results obtained from XRD measurements show a thicker cap layer is more effective to protect In composition from desorption. QCSE is enhanced and holes injection efficiency is reduced with increasing thickness of GaN cap layer. The various degrees of exciton localization effect induced by different GaN cap layer thickness explain the anomalies of PL peak wavelength and intensity for three samples. Too thin LT-cap layer will exacerbate the inhomogeneity of localization states in the InGaN QW layer, and too thick LT-cap layer will generate more defects in GaN cap layer. Therefore, optimizing the thickness of LT-GaN cap layer is a key issue to promote the uniformity and emission intensity of InGaN active layer.

## Data Availability

The datasets used and/or analyzed during the current study are available from the corresponding author on reasonable request.

## Abbreviations

MOCVD: Metalorganic chemical vapor deposition
QCSE: Quantum confined Stark effect
MQWs: Multiple quantum wells
QB: Quantum barrier
LEDs: Light-emitting diodes
LDs: Laser diodes
HT: High temperature
LT: Low temperature
EL: Electroluminescence
TDPL: Temperature-dependent photoluminescence
XRD: X-ray diffraction
FWHM: Full width at half maximum
